# Dysregulated Bradykinin: Mystery in the Pathogenesis of COVID-19

**DOI:** 10.1155/2022/7423537

**Published:** 2022-02-08

**Authors:** Aisha Tabassum, Mohammad Shahid Iqbal, Sadia Sultan, Raghad Ali Alhuthali, Deena Ismail Alshubaili, Raghad Salah Sayyam, Lama Mohammed Abyad, Ahmed H. Qasem, Ahmad F. Arbaeen

**Affiliations:** ^1^Assistant Professor of Pathology, Department of Laboratory Medicine, College of Applied Medical Sciences, Umm Al Qura University, Makkah, Saudi Arabia; ^2^Clinical Sciences Department, Fakeeh College of Medical Sciences, Jeddah, Saudi Arabia; ^3^College of Applied Medical Sciences, Umm Al-Qura University, Makkah, Saudi Arabia; ^4^Laboratory Medicine Department, Faculty of Applied Medical Sciences, Umm Al-Qura University, Makkah, Saudi Arabia; ^5^Faculty of Applied Medical Sciences, Department of Laboratory Medicine, Umm Al-Qura University, Makkah, Saudi Arabia

## Abstract

The COVID-19 pandemic is rapidly spreading, and health care systems are being overwhelmed with the huge number of cases, with a good number of cases requiring intensive care. It has become imperative to develop safe and effective treatment strategies to improve survival. In this regard, understanding the pathogenesis of COVID-19 is highly important. Many hypotheses have been proposed, including the ACE/angiotensin-II/angiotensin receptor 1 pathway, the complement pathway, and the angiotensin-converting enzyme 2/mitochondrial assembly receptor (ACE2/MasR) pathway. SARS-CoV-2 binds to the ACE2 on the cell surface, downregulating the ACE2, and thus impairs the inactivation of bradykinin and des-Arg9-bradykinin. Bradykinin, a linear nonapeptide, is extensively distributed in plasma and different tissues. Kininogens in plasma and tissue are the main sources of the two vasoactive peptides called bradykinin and kallidin. However, the role of the dysregulated bradykinin pathway is less explored in the pathogenesis of COVID-19. Understanding the pathogenesis of COVID-19 is crucial for the development of new effective treatment approaches which interfere with these pathways. In this review, we have tried to explore the interaction between SARS-CoV-2, ACE2, bradykinin, and its metabolite des-Arg9-bradykinin in the pathogenesis of COVID-19.

## 1. Introduction

Starting in December 2019, many cases of pneumonia with an unknown cause emerged in Wuhan, Hubei Province, China [1]. Later, it was established through microbe sequencing to be caused by a new coronavirus, named the severe acute respiratory syndrome coronavirus 2 (SARS-CoV-2), resulting in an international outbreak of a disease mainly involving the respiratory system, named the coronavirus disease-19 (COVID-19) [2, 3]. Since its emergence, COVID-19 infection has rapidly spread in China and many other countries around the world [1, 4]. COVID-19 was declared a pandemic in March 2020 by the World Health Organization [5].

Coronaviruses are enveloped ribonucleic acid (RNA) viruses with a nonsegmented genome that spreads and infects large number of animals as well as humans. They are named coronaviruses based on their morphology, with spherical virions and projections on the surface [6, 7]. SARS-CoV-2 has an envelope-anchored spike glycoprotein (S), which helps in the entrance of the virus into the cells by binding to a specific receptor. Once inside the cell, the virus replicates exponentially [8].

A large cohort study from China that included more than 44,000 people with COVID-19 showed that illness severity can range from mild to critical. The incidence of mild to moderate, severe, and critical illness was found to be 81%, 14%, and 5%, respectively (https://www.cdc.gov/coronavirus/2019-ncov/hcp/clinical-guidance-management-patients.html). Mild symptoms include dry cough, sore throat, and/or fever with spontaneous recovery. However, in some cases, it may lead to life-threatening situations such as pulmonary edema, severe pneumonia, diffuse pulmonary intravascular coagulopathy (DPIC), and acute respiratory distress syndrome (ARDS) [9]. Some patients have also reported nonrespiratory symptoms such as acute hepatic and cardiac injury, renal failure, and diarrhea [1]. The risk of developing a severe or critical disease is often associated with old age; comorbidities like diabetes, hypertension and cardiovascular disorders; hyperactivation of the immune system; male sex; and some other unknown factors [8].

The COVID-19 pandemic is rapidly spreading, and health delivery systems are being overburdened by the large number of patients needing intensive care, and safe and effective pharmacotherapeutic strategies are needed to improve survival [10].

The pathophysiology of COVID-19 is still not completely clear resulting in a lack of effective treatments [3]. Many complex inflammatory molecular pathways of downregulation of angiotensin-converting enzyme 2 (ACE2) in relation to SARS-CoV-2 have been proposed, like the complement system pathway, dysregulated bradykinin pathway, angiotensin-converting enzyme/angiotensin-II/angiotensin receptor 1 (ACE2/Ang-II/ATR1), and ACE2/mitochondrial assembly receptor (MasR) pathway [11, 12]. Understanding the pathogenesis of COVID-19 is crucial for the development of new effective treatment approaches which interfere with these pathways [3]. In this review, we are trying to explore the role of bradykinin and its metabolites in the pathogenesis of COVID-19.

## 2. The Kallikrein-Kinin System (KKS)

Bradykinin (BK), a linear nonapeptide, is extensively distributed in plasma and different tissues [9, 13]. Kininogens in plasma and tissue are the main sources for the two vasoactive peptides called bradykinin and kallidin [14]. Kallikreins are serine proteases that are secreted as inactive proforms and are converted to their active form extracellularly through proteolytic removal of their aminoterminal propeptide. This is a key regulatory step that occurs in both physiological function and pathological disorders to control the levels of active form of kallikreins [8, 15].

**Figure 1:**
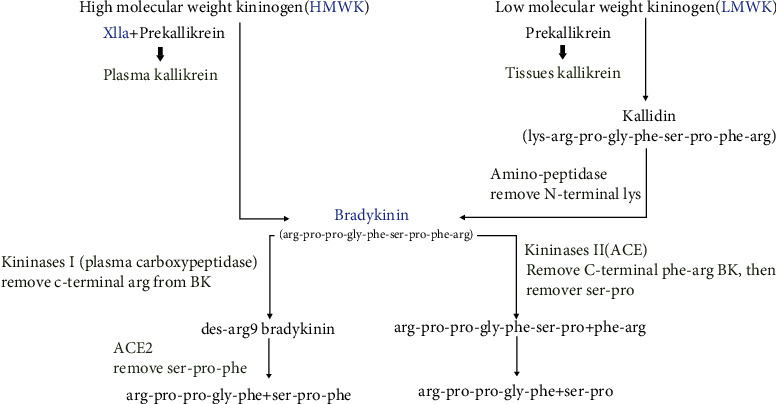
Bradykinin synthesis and degradation.

There are two general pathways for the generation of bradykinin (BK); the first one is by intracellular conversion of prekallikrein to kallikrein, and the responsible enzyme of this conversion is unknown. Tissue kallikrein is secreted by many cells, and some of them secrete it in large quantities including the lung, kidney, and glandular tissues including salivary and sweat glands and pancreatic exocrine glands, brain, intestine, and prostate ([16, 17]). This tissue kallikrein is secreted and digests the plasma substrate, low molecular weight kininogen (LK or LMWK) to provide lysyl-bradykinin (Lys-BK, kallidin) (Figure 1). Lys-BK is then cleaved by a plasma aminopeptidase to form the 9-aminoacid peptide bradykinin [14, 16, 17].

The second pathway is more complex and is carried out in the plasma, involving factor XII, plasma prekallikrein, and high molecular weight kininogen (HK or HMWK). Both prekallikrein and the coagulation factor XII circulate as a bimolecular complex with HMWK, and they compete at the same binding site but there is enough quantity of HMWK for both. When the activation continues, factor XII is then converted into its 2 active forms, and both forms convert prekallikrein to kallikrein; subsequently, kallikrein cleaves HMWK to form bradykinin (BK). Thus, bradykinin is produced both from LMWK and HMWK (Figure 1) ([16–18]).

There is enhanced production of bradykinin during inflammation as cleavage of HMWK by kallikrein is potentiated by plasmin [3].

### 2.1. Bradykinin Degradation

BK degradation is done by kininases I and II, related to the vascular endothelium. Kininase I, also known as plasma carboxypeptidase, works by converting BK or Lys-BK to produce its active metabolite des-arg9-bradykinin (des-arg9-BK or DABK). Then, kininase II, a tripeptidase, known as angiotensin-converting enzyme (ACE) also acts through a similar mechanism to inactivate bradykinin (Figure 1) [19]. Bradykinin is cleaved also by serine proteases, prolyl endopeptidases, and aminopeptidases [20, 21].

ACE inhibition results in systemic-acquired angioedema due to excessive bradykinin activating the bradykinin 2 receptor (B2R) [22]. ACE2 on the other hand does not have any action on bradykinin; instead, it inactivates des-Arg9-bradykinin (DABK), thus providing a protective effect against pulmonary edema especially in the presence of inflammation [22, 23]. Hence, ACE and ACE2 both have roles in inactivating bradykinin receptor ligands [24].

### 2.2. Bradykinin Receptors

Receptors are essential for initiating the BK action and the intracellular response. BK receptors are G-protein coupled cell surface receptors, and these are the bradykinin 1 receptor (B1R) and bradykinin 2 receptor (B2R). BK, as well as DABK, preferentially acts through these receptors, and BK acts as a ligand for B2R, while DABK is the main agonist for B1R [3, 21].

While B2R is constitutively expressed in many tissues like endothelial cells and smooth muscle cells, B1R is an inducible receptor, and expression is increased by cytokines during infections, immunopathology, and proinflammatory conditions [3, 14]. Activation of B1R and B2R through their respective ligands results in increased vascular permeability and neutrophil recruitment and hence promotes inflammation. In addition, B2R forms dimers with many of the renin angiotensin aldosterone system (RAAS) receptors that are important in regulating physiologic functions, including thrombosis risk regulation. B2R forms complexes with endothelial nitric oxide synthase (eNOS, NOS3), while B1R interacts with the cytokine-inducible nitric oxide synthase (iNOS, NOS2) [21].

### 2.3. Functions of Bradykinin

Bradykinin plays an important role in cardiovascular function, has a vasodilator effect, increases vascular permeability, and lowers blood pressure via B1 and B2 receptors. Another important inflammatory response of bradykinin through B2 receptor is pain and fever [3]. It also counterbalances the deleterious effects of Ang-II in normal conditions [25]. Bradykinin triggers cough reflex, induces bronchoconstriction, and increases airway resistance partly through B2 receptor activation. Bradykinin 2 receptors have a high affinity for bradykinin. ACE inhibitors compete with bradykinin for ACE binding sites, resulting in reduced bradykinin degradation and an increased amount of active bradykinin in the circulation and tissues causing angioedema and cough in patients on these medications [9]. Bradykinin through its action on B2R induces renin synthesis and is released by stimulating protein kinase C and nitric oxide release from the collecting duct cells. PGE2 (prostaglandin E2) produced by BK activation also releases renin. This function of BK helps in interaction between RAAS and KKS [21].

#### 2.3.1. Angiotensin-Converting Enzyme

The angiotensin-converting enzyme (ACE) is a very important component of the renin-angiotensin system (RAS), having many systemic and local effects on the cardiovascular system [26].

The human angiotensin-converting enzyme exists in two forms, ACE and ACE2, and both are zinc metallopeptidases, comprising 805 amino acids. These enzymes are present on the cell membrane as type I integral membrane glycoproteins with an extracellular N-terminus and catalytic regions that helps in the metabolism of many circulating peptides. While ACE has 2 catalytic domains, ACE2 has only one domain [27, 28].

#### 2.3.2. Tissue Distribution of Angiotensin-Converting Enzymes

ACE is distributed mainly in the vasculature and mammalian tissues, whereas ACE2 is found in many tissues, the highest levels of transcripts being found in the respiratory system mainly the lung, the cardiac tissue, and the gastrointestinal and renal system mainly proximal tubular cells. Alveolar type II cells in the lung, vascular endothelium, epithelial cells of buccal mucosa, lymphocytes, and testes express these receptors. ACE2 is expressed in all cardiac tissue components including the endothelial cells, smooth muscle cells, cardiac muscle fibers, fibroblasts, and macrophages ([27, 29]). Neurons and glial cells, tongue epithelial cells, cholangiocytes, adipose tissue, pancreatic tissue, uterus epithelial cells, ovary and breast, and the placental tissue also express ACE2 [30].

ACE2 is present in two forms, a membrane-associated form and a secreted form [29]. The membrane bound form constitutes the majority of ACE2 in the body, whereas the soluble form is present in a low concentration in circulation [27].

#### 2.3.3. Functions of ACE and ACE2

**Figure 2:**
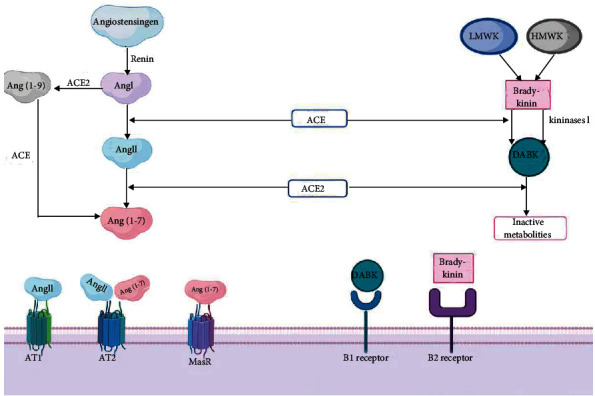
Role of ACE2 and ACE in the renin angiotensin system and kinin system.

Though structurally similar, ACE and ACE2 have different functions, and hence, the action of ACE2 is not altered by ACE inhibitors that are used in cardiovascular disorders. ACE2 acts exclusively as a carboxypeptidase, converting octapeptide angiotensin-II (Ang-II) to a heptapeptide angiotensin-(1-7) or from the decapeptide angiotensin-I (Ang-I) to nonapeptide angiotensin-(1-9) (Figure 2) [11, 27]. In contrast, ACE acts by removing the C-terminal dipeptide from Ang-I to form Ang-II, the potent vasoconstrictor, as well as hydrolyzes many other endogenous bioactive peptides [28]. Moreover, ACE2 has a regulatory effect on ACE action by reducing the amount of Ang-II through conversion of Ang-II to Ang-(1-7) (Figure 2). Other beneficial effects of ACE2/angiotensin-(1-7) include a defensive role in diabetes mellitus by reducing insulin resistance, increasing insulin secretion, and maintaining pancreatic *β* cell survival. Moreover, ACE2/angiotensin-(1-7) has a valuable role in the cardiovascular system, promotes vasorelaxation of coronary vessels, inhibits oxidative stress, and helps in the recovery of postischemic heart functions [30, 31]. Thus, ACE2 behaves as a counterregulator of the classic ACE system [11, 32].

The kallikrein-kinin system, a natural counterbalance to the renin-angiotensin system, is also regulated by ACE and ACE2. They possess degradative effects on bradykinin and des-arg9-BK, respectively.

While ACE converts bradykinin to DABK, ACE2 metabolizes DABK to biologically inactive products (Figure 2) [27, 31].

## 3. Pathogenesis of COVID-19 through Bradykinin Pathway

SARS-CoV-2 is transmitted mainly by respiratory droplets, surface contact, and contact transmission from an infected individual. Primary viral replication is supposed to occur in the mucosa of the upper respiratory tract, with further multiplication in the lower respiratory tract and gastrointestinal mucosa [1].

The first step of viral infection is its entry into the host cell [31]. ACE2 is an integral membrane protein that acts as the host-cell receptor for SARS-CoV-2. There is an increased expression of ACE2 receptors in the lungs as well as the vascular endothelial cells from patients with COVID-19 [33]. An important structural component of all types of coronaviruses is the presence of the envelope-anchored spike (S) protein, which enables the binding of the virus to its receptor on the host cell [34]. The S protein is made up of two subunits S1 and S2; S1 is responsible for the attachment and S2 for membrane fusion [30]. The spike protein binds to the ACE2 receptor on the cell membrane through its S1 subunit. The interaction between S1 and the human ACE2 receptor induces a conformational change in the S2 subunit of the spike protein, as a result of which the virus envelope fuses with the cell membrane [27]. The transmembrane proteinases, a disintegrin and metallopeptidase domain 17 (ADAM17) and transmembrane serine protease 2 (TMPRSS2), are required for virus fusion through activation of S2 [27, 30]. The SARS-CoV-2 S protein is primed by TMPRSS2, into two distinct subunits, S1 and S2, a step necessary for efficient virus replication, whereas ADAM17 cleaves ACE2 to cause ectodomain shedding [31, 34].

**Figure 3:**
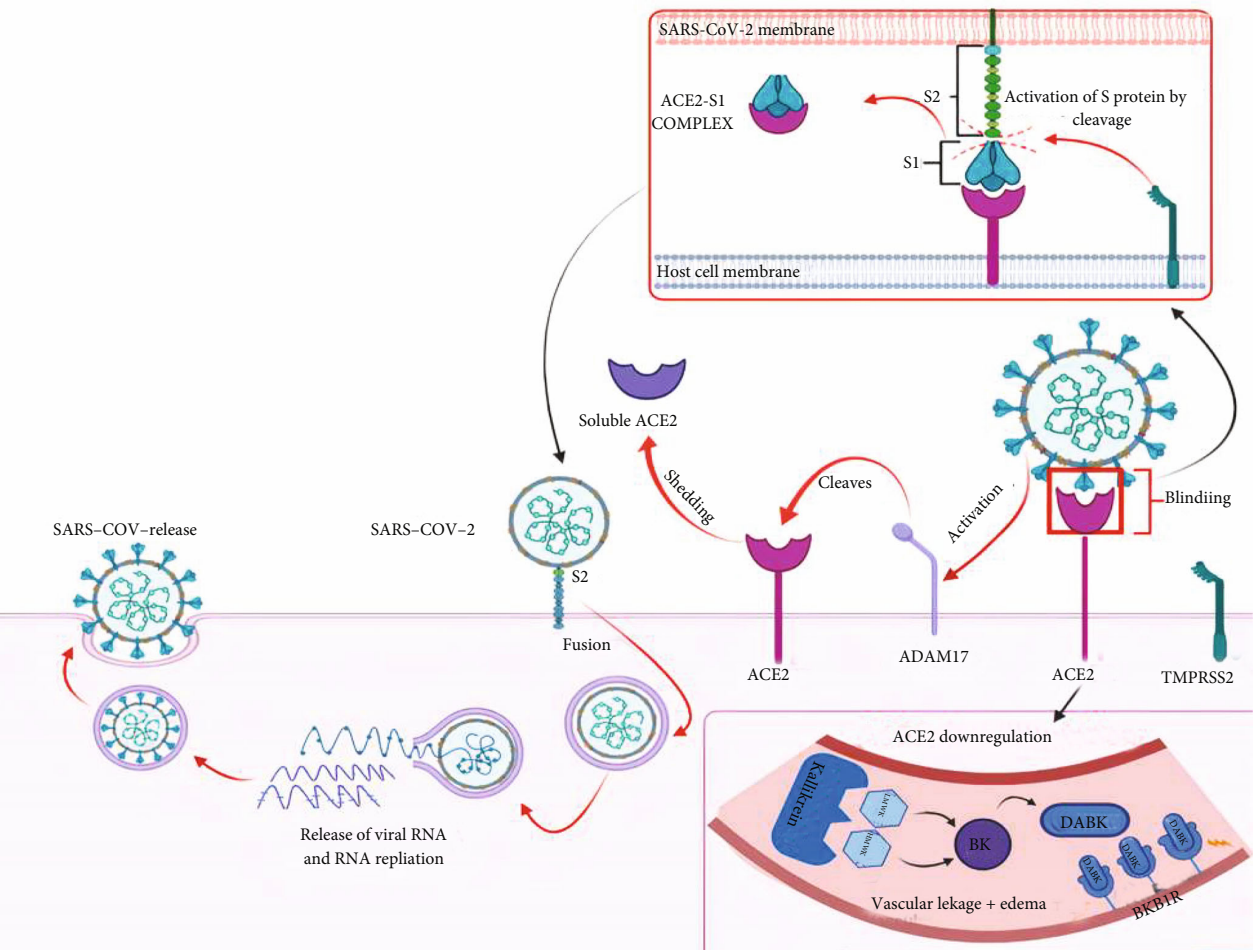
Pathogenesis of COVID-19 through bradykinin pathway.

The attachment of the virus with ACE2 results in internalization of the complex into the target cell and downregulation of the ACE2 [12]. The intact ACE2 or its transmembrane domain is internalized together with the virus, and viral RNA is subsequently released into the cytoplasm, establishing infection (Figure 3) [31].

ACE2 hydrolyzes the active bradykinin metabolite DABK into an inactive form. Downregulation of ACE2 impairs the inactivation of DABK, and hence, its signaling through B1R is enhanced (Figure 3). This results in increased vascular permeability and leukocyte extravasation to the lung. As mentioned earlier, the expression of the B1 receptor is also enhanced during inflammatory conditions, and increased levels of inflammatory mediators through the bradykinin system may increase vascular permeability, ARDS, and multiple organ failures [32].

KKS is associated with the increased vascular permeability and inflammatory response during various viral infections [21]. Formation of microthrombi is another important symptom seen in COVID-19 patients. Underlying the triggering mechanism of microthrombi could be the imbalance of the coagulation system by activation of factor XII and plasmin by the KKS [35].

Under physiological conditions, bradykinin and Lys-BK have a short half-life of around 27 seconds. Inactivation by ACE keeps the effects of bradykinin localized and prevents systemic vascular leakage and reduction in blood pressure. However, ACE inhibitors increase the half-life of bradykinin in plasma [36]. While most of the bradykinin is directly degraded, around 11% of bradykinin is converted into des-Arg9-bradykinin or DABK, its half-life being at least 10-folds higher than that of bradykinin. ACE preferentially cleaves bradykinin, whereas ACE2 cleaves DABK. Experimental reduction in ACE2 activity in the mouse models exacerbated lung inflammation through B1R activation by DABK, strongly supporting the hypothesis that downregulation of ACE2 during SARS-CoV-2 infections leads to an increased half-life of DABK and lung edema in these patients. In other words, what was claimed for angiotensin-II equally applies to DABK [36].

The B1 receptor is highly sensitive to chemical mediators like lipopolysaccharide (LPS) and cytokines such as interleukin 1 beta (IL-1B) and tumor necrosis factor alpha (TNF*α*). Interleukin 2 (IL-2), interferon gamma (IFNɣ), and epidermal growth factor (EGF) increase the rate of B1R receptor-mediated response. Activation of B1R enhances the chemotaxis of neutrophils to tissue by the release of chemokine C-X-C motif chemokine 5 (CXCL5). COVID-19-induced downregulation of ACE2 activity accompanied with increased activity of DABK results in the accentuation of this inflammatory cascade, leading to increased cytokine release and possibly explains the cytokine storm seen in COVID-19 patients [8, 12, 22]. The cytokine storm if not controlled can lead to cytokine leakage into the bloodstream and attack other ACE2-expressing cells in organs like the kidney and heart and also causes a systemic inflammatory response in the body affecting various body systems, like the hepatic system, gastrointestinal tract, and even CNS [31]. In essence, this overproduction of cytokines is the immune system attacking the patient's body leading to multisystem organ failure and death [34].

Many clinical features of COVID-19 that are characteristic of the disease can be explained by the activation of the bradykinin pathway. Dry cough, being one of the most common symptoms, is ascribed to increased bradykinin activity. This increased level of bradykinin exceeds the capacity of aminopeptidase as an alternative catalyst for its degradation. Aminopeptidase is a zinc-dependent enzyme with a low reserve, leaving the bradykinin system unabated. Hence, the effects of zinc supplements have been proposed to increase the levels of aminopeptidase. Drop in oxygen saturation, bronchospasm, and vascular leakage can all be explained by bradykinin activity [9]. Some patients also present with atypical symptoms such as headache, abdominal cramps, diarrhea, and other gastrointestinal symptoms like nausea and loss of taste, dizziness, anosmia, and dysgeusia, explained by the fact that SARS-CoV-2 target many ACE2-expressing tissues [9, 37]. Excess of bradykinin can result in hypokalemia leading to arrhythmia and sudden cardiac death, and these events have been reported in severe COVID-19 patients [37].

Furthermore, critically ill patients' vasopressors are required to stabilize the blood pressure, and it is a well-known fact that an increase in bradykinin level lowers the blood pressure. Anosmia and/or dysgeusia reported by some COVID-19 patients is a well-known side effect of ACE inhibitors. Extreme thirst, an uncommon symptom of COVID-19 patients, is often seen with elevated BK and ACE inhibitor levels. Confounding factors might coexist, and therefore, further evaluations are needed [10].

The potential risk of SARS-CoV-2 infection depends on the ACE2 expression in various tissues. Organs can be classified based on the ACE expression as high risk and low risk types with >1% proportion of the ACE2 expression and <1% proportion of ACE2-positive cells, respectively. Thus, the high-risk group includes the lower respiratory tract (2%), lung (>1%), heart (>7.5%), ileum (30%), oesophagus (>1%), kidney (4%), and bladder (2.4%), and the low-risk group includes the stomach and liver [30]. The expression of ACE2 in type II alveolar epithelial cells is very high, which might explain the severe alveolar damage seen after SARS-CoV-2 infection. However, ACE2 is also expressed in the kidney, heart, tongue, and gastrointestinal system and thus explains the nonrespiratory symptoms in COVID-19 patients [34].

Lower expression of ACE2 in the nasal epithelium and bronchial epithelial cells in children and young adults explains lower incidence of SARS-COV-2 infection and the nonrespiratory COVID-19 symptoms in them [30]. It is considered that the ACE2 expression is 3-folds higher in male as compared to female lung samples, explaining the higher fatality rate of COVID-19 in male patients. Pregnant women are at a higher risk of COVID-19 infection because ACE2 also expresses in the female reproductive system [30, 31].

The ACE/ACE2 ratio has an important role in different diseases including IgA nephropathy, diabetes mellitus, subtotal nephrectomy, and hypertension. A rise in the ACE/ACE2 ratio as seen during COVID-19 infection might result in the development of kidney damage [32].

Analyzing plasma levels of BK and DABK in patients with COVID-19 especially those with respiratory complications might help support the hypothesis of the ACE2/bradykinin pathway [10]. Gene expression studies in bronchoalveolar lavage (BAL) specimens from COVID-19 patients have shown the upregulation of kallikreins and kininogen resulting in increased bradykinin synthesis. The expression of the B1 and B2 receptors was increased by 2945- and 207-folds, respectively. The expression of kininogen and kallikreins is undetected in controls but expressed in COVID-19. While the gene expression of ACE was reduced 8-fold, resulting in enhanced activity of bradykinin, on the other hand, downregulation of ACE2 results in reduced degradation of DABK. This in combination with the increased expression of the B1 and B2 receptor can lead to the bradykinin storm [16, 37].

### 3.1. Therapeutic Approach to ACE2/Bradykinin Pathway in COVID-19 Patients

The mainstay of the present treatment for COVID-19 patients is mainly supportive. Despite applying intensive supportive regimens to control the infection, respiratory failure due to the ARDS remains the main cause of mortality. Thus, understanding the exact pathophysiology underlying the disease process becomes of utmost importance for designing effective treatments [9]. The role of the dysregulated bradykinin system in the pathogenesis of COVID-19 should be explored to provide effective treatment. It can be suggested that the severity of COVID-19 and its high mortality are because of vascular problems as a result of activation of B1 and B2 receptors ([13, 38]).

Many steps in the kallikrein-kinin pathway might be the potential target for COVID-19 treatment, like the blockade of tissue kallikrein activity, hence reducing the production of kinins or enhancing the kinin degradation by the use of recombinant active enzymes such as ACE2 or by blocking the bradykinin receptors B1 and B2. Blocking B1 and B2 receptor signaling would be the most potent and effective among all these steps.

Icatibant is a peptide B2 receptor antagonist which is available in the US and Europe for the treatment of hereditary angioedema in adults, adolescents, and children. It is a synthetic decapeptide with a structure like bradykinin [18, 22]. It is safe and effective, with rare side effects and adverse reactions in patients with hereditary angioedema [38, 39]. A study showed that the use of icatibant to treat allergic rhinitis reduced grass pollen antigen-induced hyperresponsiveness to histamine, by inhibiting interleukin-8 (IL-8) release. IL-8 is implicated in acute lung injury and respiratory distress in COVID-19 patients, and this further supports the empirical use of icatibant in the treatment of unremitting respiratory distress [10].

Lanadelumab is a monoclonal antibody against the plasma kallikrein, which cleaves HMWK kininogen into bradykinin, also involved in the coagulation and induction of the complement system. Lanadelumab is in use for the treatment of angioedema with no significant reports of adverse and severe events. In COVID-19 patients, lanadelumab can block the upstream axis that leads to kinin formation. It can be used as an adjuvant to the antiviral therapy, bringing down the inflammatory and coagulation storm besides the complement system in SARS-CoV-2-infected patients [8, 22].

Drugs that antagonize the action of tissue kallikrein and the B1 receptors of bradykinin are still at the research level and are still not approved for clinical use, but such agents are needed and will be useful in the treatment of COVID-19 cases [16].

## 4. Conclusion

Interaction between SARS-CoV-2, ACE2, bradykinin, and DABK very well explains the acute respiratory distress syndrome, cytokine storm, multiorgan failure, and all the common clinical manifestations experienced by the COVID-19 patients. However, this hypothesis has not been proven clinically; thus, it is necessary to develop clinical studies to test this hypothesis. Many drugs such as icatibant and lanadelumab which are already available and are in clinical use for patients with angioedema can benefit the COVID-19 patients as well. This will help in breaking the vicious cycle created by the dysregulation of the bradykinin pathway in these patients, reducing morbidity and mortality and bringing a favorable outcome.
